# Bibliometric analysis of global research trends on small-cell lung cancer (2012–2021)

**DOI:** 10.3389/fonc.2022.955259

**Published:** 2022-10-06

**Authors:** Kai Wang, Han Zhang, Xin Li, Yun Ding, Jiuzhen Li, Zixiao Wang, Xin Liu, Shuai Sun, Daqiang Sun

**Affiliations:** ^1^ Clinical School of Thoracic, Tianjin Medical University, Tianjin, China; ^2^ Department of Thoracic Surgery, Tianjin Chest Hospital of Tianjin University, Tianjin, China

**Keywords:** small cell lung cancer, immunotherapy, bibliometric analysis, research trends, hot spots

## Abstract

**Background:**

Small-cell lung cancer (SCLC) is a recalcitrant tumor with a poor prognosis. With the rise of SCLC research in the past decade, this study aims to analyze the foundation and frontiers of SCLC research through bibliometric analysis.

**Methods:**

Relevant publications from the Web of Science Core Collection were retrieved on January 3, 2022. R package bibliometrix and EXCEL2019 were used to analyze quantitative variables. Bibliometric mapping was constructed by VOS viewer and CiteSpace software to visualize citation, co-authorship, co-occurrence, and co-citation analysis of countries/regions, organizations, authors, references, and keywords.

**Results:**

A total of 2,361 publications related to SCLC were identified with the total amount of articles steadily increasing, where China is the most productive country with 859 papers. Scholars and organizations from the United States, China, and Europe are primary sources of this research, among which the University of Texas MD Anderson Cancer Center made the most contribution to the field with 122 papers. *Lung Cancer* published the highest number of SCLC-related articles with a total of 121, while *the Journal of Thoracic Oncology* received the most citations totaling 3,098. Rudin, Charles M., and Sage, Julien are the most creative author. Leora, Horn, 2018, *New Engl J Med* and Rudin, Charles M., *Nat Genet*, 2012 can be categorized as classic literature owing to their high citations or strong sigma value. “Heterogeneity & Subtypes” and “Immunotherapy” may be the new frontiers in the SCLC domain.

**Conclusion:**

Research on SCLC showed an upward trend based on the current global situation. Moreover, the current scope of collaboration in SCLC research is chiefly regional, which should further focus on transnational cooperation in the future. More attention should be devoted to “Heterogeneity & Subtypes” and “Immunotherapy”, which will be the hotspots in future research.

## Introduction

Lung cancer, one of the most malignancies with the highest fatality rate in the world, where there were about 2.2 million new cases and 1.79 million new deaths of lung cancer in 2020 that accounting for 11.4% of all tumor cases and 18% of all tumor deaths, respectively according to GLOBOCAN 2020 (1). The 2 major histopathological types of lung cancer are small-cell lung cancer (SCLC) and non-small-cell lung cancer (NSCLC). SCLC, categorized into two clinical stages including limited and extensive stage, is generally diagnosed in smokers (2), whose treatment remains a formidable challenge for decades. Surgery is merely a choice for patients in limited-stage SCLC (LS-SCLC) (3), while chemotherapy has been the only standard treatment option for SCLC for the past 3 decades until the incorporation of first-line immunotherapy, yet most patients relapse and rarely survive for more than two years (3, 4). Despite the continuous innovation and development of diagnosis and treatment technology, there is no significant progress in the 5-year overall survival rate of SCLC patients (5, 6), with a high mortality rate owing to that the most cases are already in the advanced stage when diagnosed, and the treatments methods are only palliative (7). Therefore, it is imperative to conduct related research on SCLC, which can not only benefit the patients suffering from SCLC but also promote the scientificity and effectiveness of lung cancer treatment.

For now, SCLC is still a fatal disease and remains a challenge in modern medical research with its underlying disease mechanisms unclear, yet the field of SCLC has stagnated since 1997. It was not until 2012 that the United States Congress passed the statute proposed by the United States National Cancer Institute to designate SCLC as a “recalcitrant cancer”, which caused a worldwide surge in SCLC research (8). In recent years, researchers have conducted positive and beneficial explorations in basic and clinical research of SCLC, and perception of the literature and attention to the frontiers of SCLC research field has evolved over time as discoveries continue to emerge (9). According to the previous reviews, the application of chemotherapy in SCLC was once the main content. With the rise of the concept of precision therapy and immunotherapy, changes have taken place in the SCLC research. However, there is no systematic bibliometric analysis to summarize the current research status.

Bibliometric analysis is an effective method to evaluate publications on a precise topic, which combines mathematical and statistical methods with data visualization to establish the overall knowledge structure, development trends, and research priorities in a specific domain (10, 11). With continuous updates of bibliometric techniques, visualization software and websites such as VOS viewer and CiteSpace, enable researchers to create science mapping conveniently (12, 13). To date, there has been no systematic study of global research trends in SCLC, making it imperative to investigate the overall situation of SCLC research to facilitate researchers in tracking research progress. The current research question is that the diagnosis and treatment of SCLC are in a period of rapid change, but there is a research gap in the absence of systematic analysis to summarize and analyze this trend. Once a systematic study is carried out in the form of bibliometric analysis, it will increase researchers’ understanding of the frontiers of SCLC domain and improve the efficiency of scientific research (14, 15). Hence, this study aims to analyze the current status and future hotspots of the SCLC domain during this active decade by bibliometric methods and fill this research gap.

## Methods

### Data source and retrieval strategies

**FIGURE 1:**
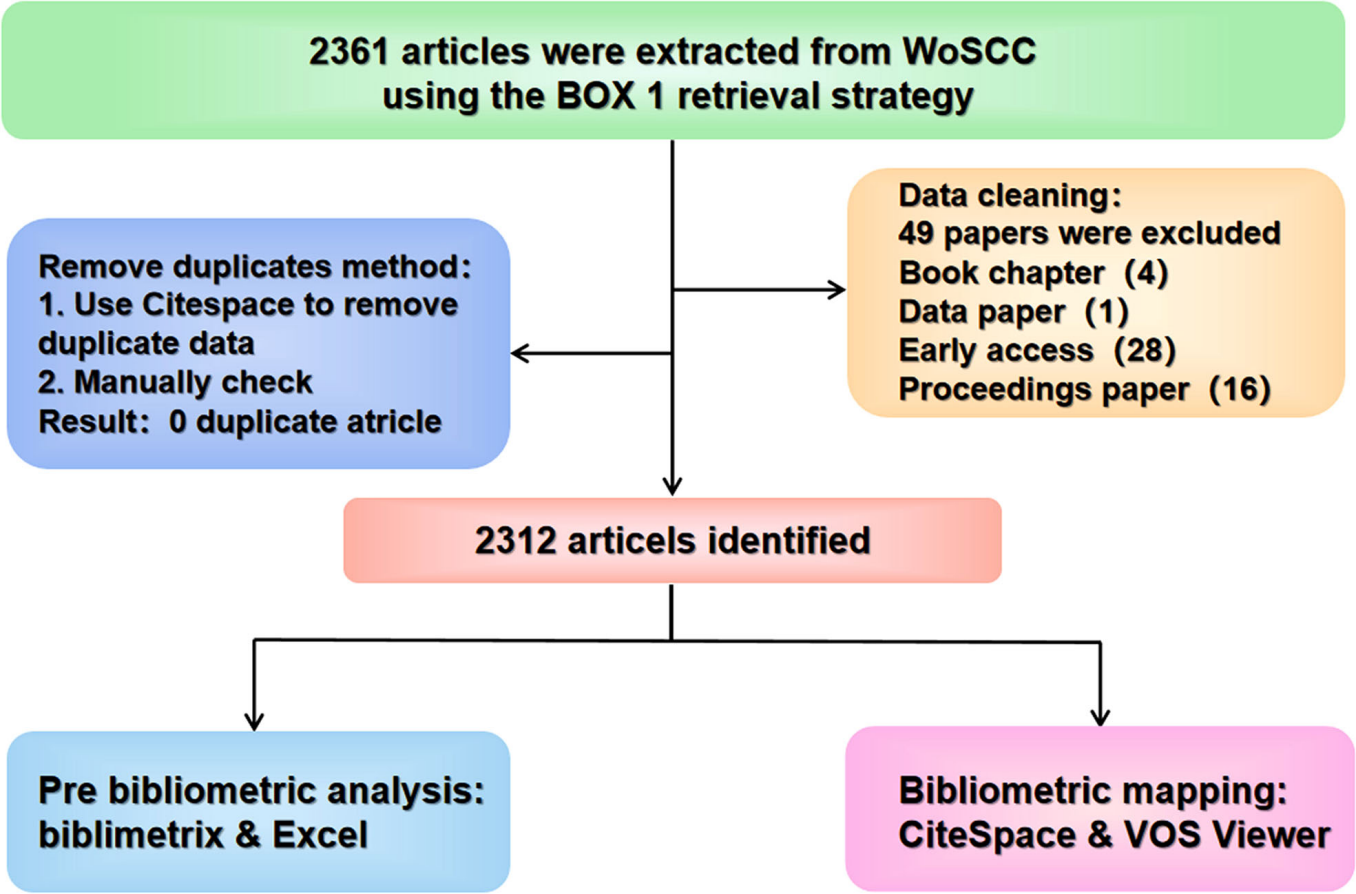
Flowchart for literature selection.

The Science Citation Index Expanded (SCI-Expanded) of the Web of Science Core Collection (WoSCC) database (https://wcs.webofknowledge.com) was chosen as the data source. WoSCC was one of the largest databases of scholarly literature, and was regarded as the most commonly used database for bibliometric research. The inclusion and exclusion criteria of SCLC are used to control the data sources of this study. Two independent authors (Wang & Zhang) conducted the search using followed retrieval strategy (**Box 1**) on January 3, 2022. This study only includes articles published in English, while others are excluded. In addition, other types of lung cancer were eliminated through Query formulation design. The document types were limited to “Articles” only, with the search period restricted from 2012 to 2021, where the full records and cited references of the retrieved documents were exported as plain text files. This study relied the extracted documents as a basis to prevent the changes in the number of papers over time from affecting the results. After the data extraction, the two researchers mentioned before examined all the documents to ensure their relevance to the research topic. The data cleaning and literature selection methods are shown in **Figure 1**.

**BOX 1 | Query formulation**

TI=(“SCLC” OR “small-cell lung cancer” OR “small-cell lung carcinoma”) NOT TI=(“NSCLC” OR “non-small cell lung cancer” OR “non-small cell lung carcinoma”) NOT TI=(“LUAD” OR “lung adenocarcinoma”) NOT TI=(“SqCLC” OR “LUSC” OR “lung squamous cell carcinoma” OR “squamous cell lung cancer”) NOT TI=(“large cell neuroendocrine carcinoma” OR “large cell carcinoma”) NOT TI=(“atypical carcinoid tumor” OR “typical carcinoid tumor”)

Document types: Articles

Languages: English

Timespan: 2012-01-01 to 2021-12-31 (Publication Date)

### Data visualization and analysis

Literature data extracted from WoSCC were imported into the R package bibliometrix (16) for preliminary bibliometric analysis, and information such as the number of published articles, citations, H-index, authors, countries, and journals was subsequently exported and recorded in EXCEL. CiteSpace V5.8.R3 (Drexel University, Philadelphia, PA, the United States) and VOS viewer 1.6.17 (Leiden University, the Netherlands) were employed to construct visualized maps of scientific literature (12, 13). Common methods of bibliometric analysis include citation analysis, co-authorship analysis, co-occurrence analysis, and co-citation analysis (17), with countries/regions, journals, organizations, authors, and keywords considered as the research objects of analysis. Citation analysis is carried out with respect to the citation of research objects and their relationship (18). Co-authorship analysis concentrates on the cooperation among the participants of the study. Keyword co-occurrence analysis refers to analysis of keyword plus generated by a special algorithm, which is derived from the title, abstract, and keywords, and is more comprehensive than the keywords provided by the author (19). Co-citation analysis can offer the association between the study subjects by the number of references cited together. Through clustering the co-cited articles, a comprehension of the topology of publications was constructed (20). A researcher’s H-index refers to that he had most H papers cited at least H times respectively, and H-index can relatively accurately reflect a researcher’s academic achievements.

### CiteSpace software analysis

In this study, CiteSpace V5.8 R3 (13) was mainly employed to create co-citation analysis mapping, journal dual-map, and reference timeline mapping, which all consist of nodes and links, with different meanings in different analysis methods (21). In co-citation analysis mapping, the node size depends on the number of co-cited references, while the links between nodes represent the co-citation relationships. Cluster analysis, a method for summarizing the same type of nodes so as to visually separate different research types, which is needed when node types cannot be classified directly (22). In this study, cluster analysis served to summarize the research progress of the reference timeline mapping. In addition, the degree of centrality index showed by CiteSpace software, is another vital indicator used to indicate the among nodes (articles) and evaluate the significance of nodes in a definite network (23). Burstness can reflect emerging academic trends and new topics, predict frontier research directions, and reveal potential hotspots in a field. Sigma is a combination of centrality and burstness, which can be used to identify novel literature and innovative topics, with the calculation formula as follows: Sigma = (centrality + 1) * burstness. Z-score and F-score are used to re-adjust or standardize the citation data and can be used to identify major citation paths in the Dual-map.

CiteSpace parameters are set as follows: Time slicing from 2012-JAN to 2021-DEC, Years per slice 1 or 2, Term source including title, abstract, author keywords, and keywords plus, Node type select from author, country, journal, institution, and keyword, Selection criteria: top 20-50, Pruning: pathfinder plus pruning sliced networks.

### VOS viewer software analysis

The Java program VOS viewer 1.6.17 (12) is a free bibliometric visualization software for citation analysis, co-authorship analysis, and keyword co-occurrence analysis in this study. The nodes and lines in the VOS viewer maps stand for the weights and associations of the study objectives including countries, journals, organizations, authors, and keywords, where the scientific mapping generated by the VOS viewer automatically groups the nodes into different colors by clusters with total link strength (TLS) applied to quantify the value of links using different meanings, such as the number of references in citation analysis (24).

The settings of the VOS viewer are as follows: Create a map based on bibliographic data (WOS files), Type of analysis (co-authorship, co-occurrence, citation), Unit of analysis (authors, organizations, countries, sources, keywords plus), Chose threshold (filter according to the output results, Countries/Regions: 1,000 citations; Organizations: 20 articles; Journals: 250 citations; Authors: 700 citations; Keywords: 30 co-occurrences).

## Results

### Global publishing trends

**FIGURE 2:**
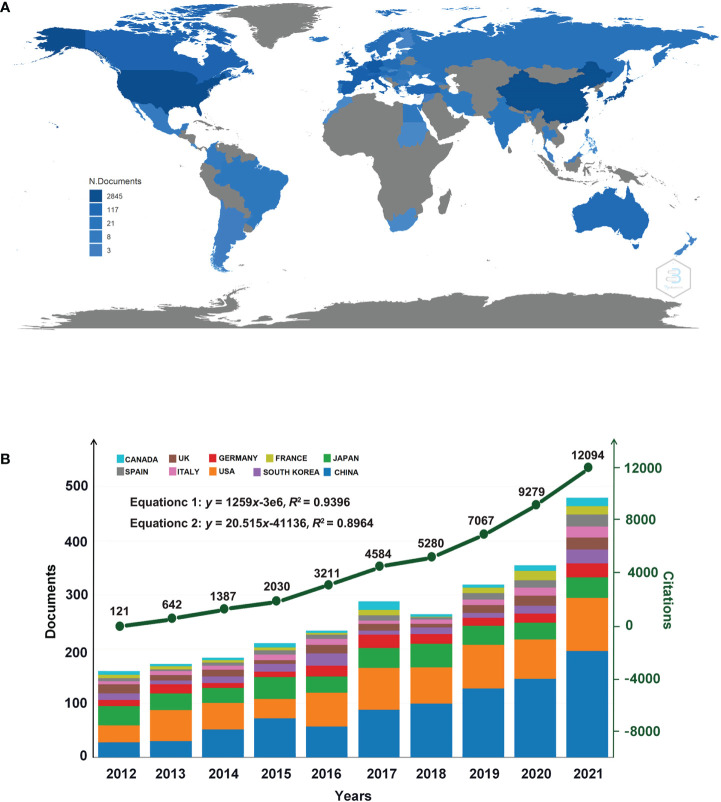
**(A)** Geographical distribution of global articles of SCLC. The darker the national color block on the map, the more SCLC-related articles are published. **(B)** Dynamics of publications and total citations in the top 10 countries in the past 20 years. The number of articles and citations was on the rise. Equation 1 can predict the annual citations, while Equation 2 can predict the annual documents.

A total of 2,361 articles from the WoSCC database were extracted, and 2,312 articles were finally confirmed to be eligible after cleaning the data. As shown in **Figure 2B**, a slow increase in the number of global publications was observed, where the number of articles published in 2021 was almost 2.3 times that of 10 years ago with the greatest growth occurring in 2021, up by 88 articles than in 2020, given that the amount of published articles remained stable for several years. The total number of citations was 44,812 times, with an average of 18.98 times per article. Over the past decade, SCLC-related articles experienced highly significant growth in citations (**Figure 2B**). The equation in **Figure 2B** can predict the number of citations or articles yearly, and the R-square is 94% and 89.64% respectively. Besides, a linear regression about the annual growth of publication and citation number is shown in **Supplementary Figure 1**. The average citation per document and the average total citation per year are shown in **Supplementary Figure 2**.

### Analysis of countries/regions

Researchers from 48 countries have conducted SCLC studies and published related articles, among which China was the most prolific country with 859 articles, followed by the United States with 479 articles and Japan with 296 articles **(** **Table 1** **)**. However, there were only 60 multiple-country publications (MCP) in China, which is less than 141 MCP in the United States, indicating that the international cooperation in China needs to be strengthened. In term of citations, the United States come in first place with 16,396 citations, while China is second with a total of 8,166 citations, and Japan with 4,295 citations **(** **Table 1** **)**. The geographical distribution of global articles is displayed on a world map in **Figure 2A**, from which can be seen that Asia, North America, and Europe are major sources of research of the most publications. **Figure 2B** illustrates the dynamics of the number of publications and total citations (TC) in the top 10 countries in the past 10 years with the number of articles published in China increasing annually, indicating the intense vitality of research in this field.

**Table 1:** The top 10 productive countries with publications of SCLC.

Rank	Country	Articles	TC	SCP	MCP	MCP Ratio
1	China	859	8166	799	60	0.0698
2	The United States	479	16396	338	141	0.2944
3	Japan	296	4295	280	16	0.0541
4	Korea	117	1759	106	11	0.094
5	Germany	103	3799	67	36	0.3495
6	United Kingdom	58	2811	35	23	0.3966
7	Italy	56	922	46	10	0.1786
8	Turkey	46	208	43	3	0.0652
9	Spain	43	1947	22	21	0.4884
10	Canada	41	576	25	16	0.3902

TC, Total citations; SCP, Single-country publication; MCP, Multiple-country publication; MCP Ratio, MCP/Articles.

**FIGURE 3:**
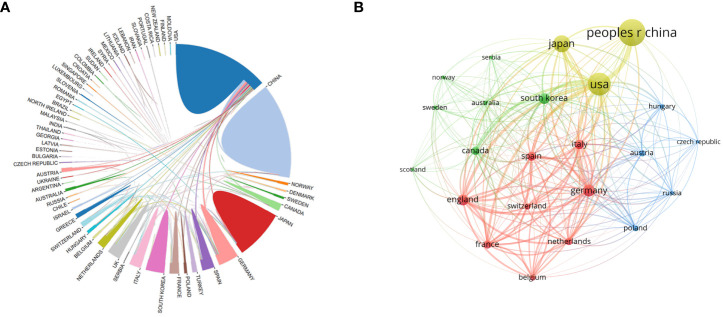
**(A)** The collaboration of countries. Each country is represented by a color block, and larger blocks indicate a higher number of publications. The line between the blocks represents the cooperation between countries. **(B)** Citation relationships between countries. China, the United States, and Japan are the major contributors to SCLC.

The collaboration of countries is shown in **Figure 3A**, where the United States is the center of cooperation with the closest collaboration between China and Germany. With the minimum citation of 1,000 as the screening criteria, a total of 23 countries were screened and the citation relationships among countries were mapped by the VOS viewer (**Figure 3B**). The largest node in **Figure 3B** signifies China, implying that Chinese researchers published the most SCLC-related articles.

### Analysis of organizations

**FIGURE 4:**
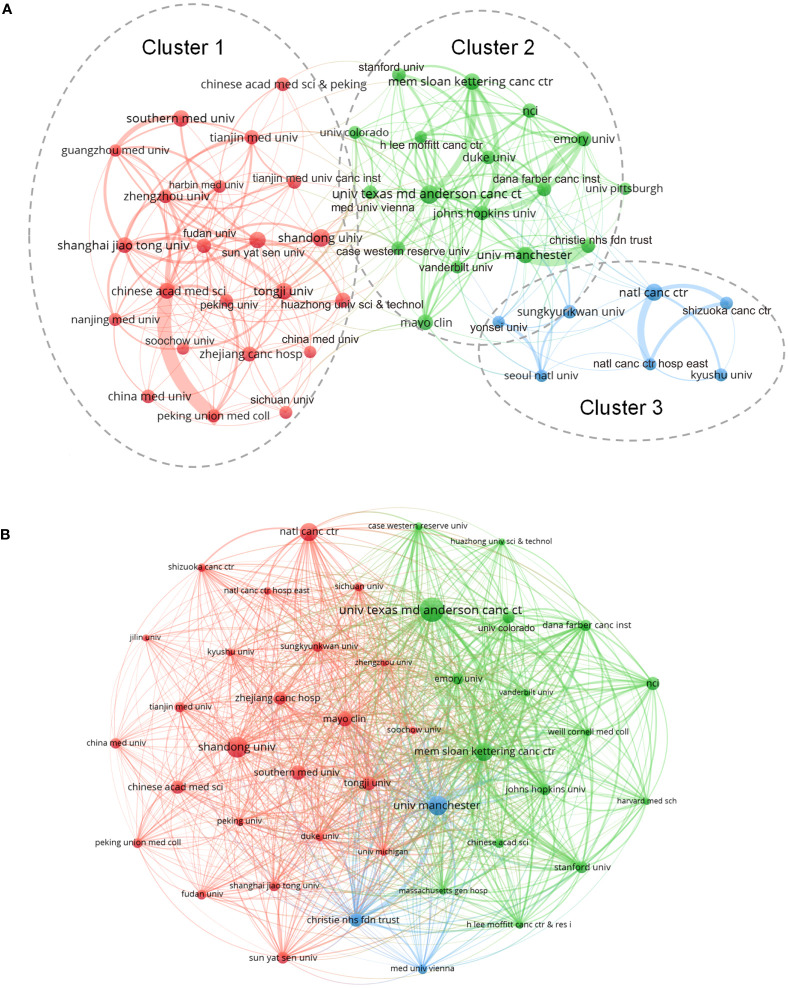
**(A)** Co-authorship relationships between organizations. Cluster 1; Chinese organizations (Red node cluster); Cluster 2; European and American organizations (Green node cluster); Cluster 3; Japanese and Korean organizations (Blue node cluster). **(B)** Citation relationships between organizations. Top3 TLS was: The Chinese Academy of Medical Sciences (TLS=68), The University of Texas MD Anderson Cancer Center (TLS=65), and Memorial Sloan Kettering Cancer Center (TLS=55).

A total of 2,996 organizations were involved in the domain, where the University of Texas MD Anderson Cancer Center ranked the top with 122 published papers, next to Memorial Sloan Kettering Cancer Center with 104 papers and Shandong University with 102 papers (**Table 2**). The co-authorship analysis using “organizations” as node settings showed that the partnerships can be divided into three groups, namely Chinese organizations represented by red node cluster, European and American organizations denoted by green node cluster, and Japanese and Korean organizations marked by blue node cluster (**Figure 4A**), with the University of Texas MD Anderson Cancer Center exhibiting the highest centrality which indicates that it is at the center of cooperation. The citation relationship between organizations is shown in **Figure 4B**, of which only 43 organizations with more than 20 articles were drawn into the VOS viewer map. Besides, the top three organizations in terms of TLS were: The Chinese Academy of Medical Sciences (TLS=68), The University of Texas MD Anderson Cancer Center (TLS=65), and Memorial Sloan Kettering Cancer Center (TLS=55).

**Table 2:** The top 10 institutes that contributed to articles about SCLC.

Rank	Organizations	Country	Articles	X% of total
1	The University of Texas MD Anderson Cancer Center	United States	122	5.28
2	Memorial Sloan Kettering Cancer Center	United States	104	4.50
3	Shandong University	China	102	4.41
4	University of Manchester	United Kingdom	94	4.07
5	Southern Medical University	China	90	3.89
6	Tongji University	China	90	3.89
7	Sun Yat-Sen University	China	81	3.50
8	Stanford University	United States	74	3.20
9	National Cancer Center	Japan	67	2.90
10	Sungkyunkwan University	South Korea	66	2.85

### Analysis of journals

**FIGURE 5:**
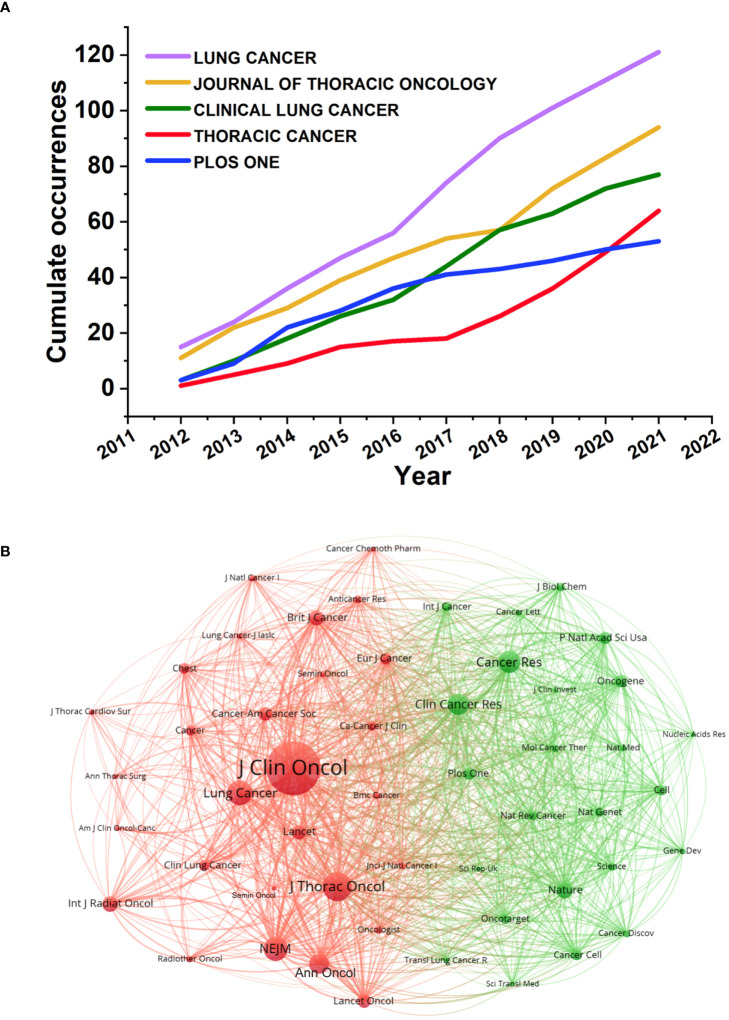
**(A)**Articles dynamics of the top 5 journals. The number of articles published in the 5 journals in the figure is increasing year by year. **(B)** Co-Citation relationships between journals. As the largest node in the graph, *the Journal of Clinical Oncology* has the most co-citations and TLS.

SCLC articles have been published in 529 journals over the past 10 years so far, among which the most influential journal is *the Journal of Thoracic Oncology* (IF2020 = 15609, JCR 2020=Q1) with 32 H-index, a high-level oncology journal. The top 10 journals ranked by the number of publications are shown in **Table 3**, from which can be concluded that *Lung Cancer* (IF2020 = 5705, JCR 2020=Q1) published most SCLC articles with 121 publications, accounting for 5.23% of all SCLC manuscripts, followed by *Journal of Thoracic Oncology* (IF2020 = 15609, JCR 2020=Q1) and *Clinical Lung Cancer* (IF2020 = 4785, JCR 2020=Q2). There were 22 journals with an H-index above 10, and the top three journals are *Journal of Thoracic Oncology* (25), *Lung Cancer* (26), and *Oncotarget* (23) respectively. In particular, *Lung Cancer* has shown sustained attention in the field of SCLC with more than 10 articles published every year (**Figure 5A** **)**.

**Table 3:** The top 10 productive journals related to SCLC.

Rank	Journals	Country	Articles	H-index	TC	IF 2020	JCR 2020
1	*Lung Cancer*	Netherland	121 (5.23%)	27	2118	5.705	Q1
2	*Journal of Thoracic Oncology*	United States	94 (4.06%)	32	3098	15.609	Q1
3	*Clinical Lung Cancer*	United States	77 (3.33%)	17	997	4.785	Q2
4	*Thoracic Cancer*	China	65 (2.81%)	10	339	3.5	Q2
5	*Plos One*	United States	53 (2.29%)	20	1191	3.24	Q2
6	*Oncotarget*	United States	46 (2.00%)	23	1091	–	–
7	*Oncology Letters*	Greece	43 (1.86%)	9	354	2.967	Q4
8	*Anticancer Research*	Greece	39 (1.69%)	10	299	2.48	Q4
9	*Medicine*	United States	34 (1.47%)	9	217	1.889	Q3
10	*Frontiers in Oncology*	Switzerland	32 (1.38%)	5	46	6.244	Q2

“–” represents the journal have been eliminated by SCI database.

**FIGURE 6:**
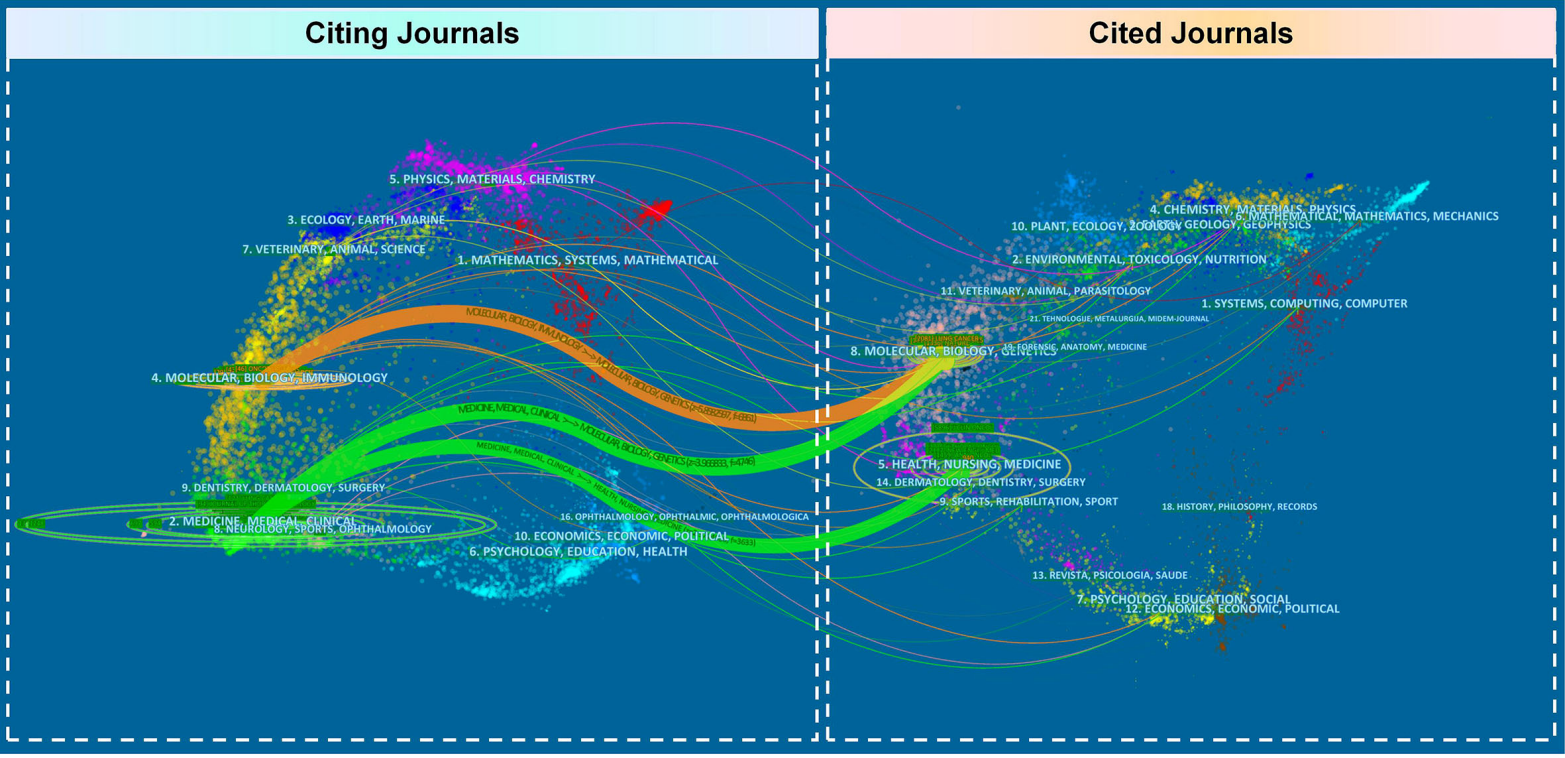
Dual-map overlay of journals produced by CiteSpace. The color bar on the left side of the figure represents the field of the citing journals, the color bar on the right side speaks for the domain of the cited journals, and the connecting line between them represents the citation relationship.

The result of co-citation analysis of journals was presented as a network map with 52 nodes and 250 links (**Figure 5B**). The top three journals with the largest TLS were *the Journal of Clinical Oncology* (TLS=161,785), *Journal of Thoracic Oncology* (TLS=90,308), and *The New England Journal of Medicine* (TLS=67,704). **Figure 6** shows a dual-map overlay of the journals on SCLC research. There are three citation paths from the “Citating Journals” part to the “Cited Journals” part, which represent the flow of research and the intersection of domains with their annotations at the starting point and ending point of the path referring to the research field of related journals. As a result, the research flow in the SCLC field is mainly as follows: (i). Molecular, Biology, Immunology → Molecular, Biology, Genetics (Z=5.86, F=6,861); (ii). Medicine, Medical, Clinical → Molecular, Biology, Genetics (Z=4.00, F=4,746); (iii). Medicine, Medical, Clinical → Health, Nursing, Medicine (Z=2.98, F=3,633).

### Analysis of authors

In **Table 4**, the top 10 authors who received the most citations in the field of SCLC are presented, who have produced 155 articles in total, accounting for 6.70% of all retrieved documents. Rudin, Charles M. from the Memorial Sloan Kettering Cancer Center who published 36 articles and gained 3,654 citations, ranked first among all authors, the second place was taken by Sage, Julien from the Stanford University with 17 articles and 3,065 citations, and Leora, Horn from the Vanderbilt University ranked third who has worked out 15 articles and was cited 2,580 times. The top three cited authors are all from different research organizations in the United States, which indicated the active engagement of American researchers in this field.

**Table 4:** The top 10 high-yield authors measured by citation.

Rank	Author	Country	Main affiliation	TC	H-index	Articles
1	Rudin, Charles M.	United States	Memorial Sloan Kettering Cancer Center	3654	26	36
2	Sage, Julien	United States	Stanford University	3065	15	17
3	Leora, Horn	United States	Vanderbilt University	2580	13	15
4	Thomas, Roman	Germany	University of Cologne	2449	9	11
5	George, Julie	Germany	University of Cologne	2366	9	11
6	Dive, Caroline	United Kingdom	University of Manchester	2292	15	20
7	Martin, Peifer	Germany	University of Cologne	2248	7	7
8	Havel, Libor	Czech Republic	Thomayer Hospital	2222	6	6
9	Reck, Martin	Germany	German Center Lung Research	2158	15	19
10	Nishio, Makoto	Japan	Japanese Foundation for Cancer Research	2113	12	17

**FIGURE 7:**
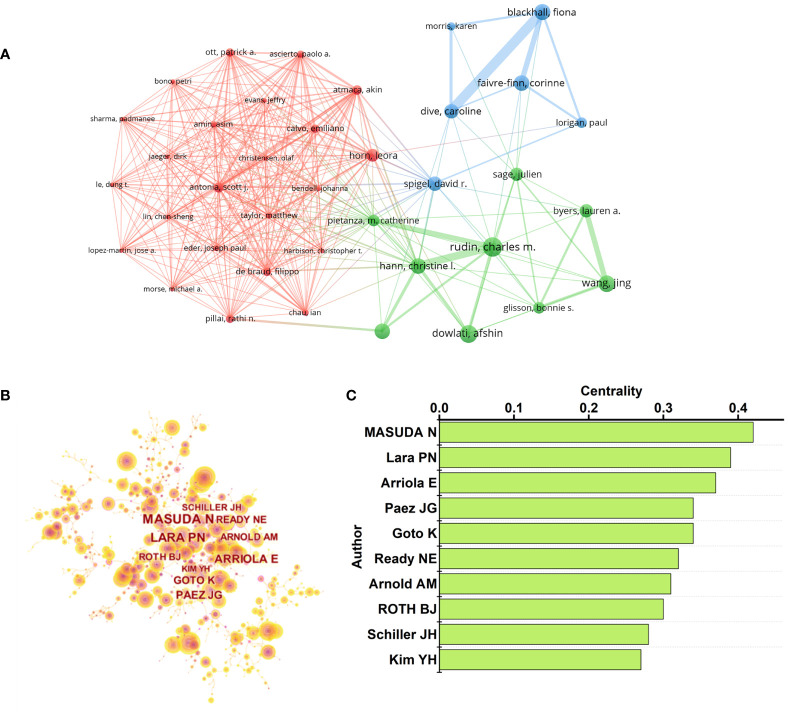
**(A)** Co-authorship relationships between authors. Top3 TLS was: Antonia, Scott J. (TLS=42), Atmaca, Akin (TLS=42), and Hann, Christine L (TLS=40). **(B)** Co-Citation relationships between authors. **(C)** The top 10 authors of centrality in **Figure 7B**.

As shown in **Figure 7A**, Antonia, Scott J., Atmaca, Akin, and Hann, Christine L. are located in the center of the collaboration map with TLS of 42, 42, and 40, respectively, demonstrating that the outputs of these 3 authors are the key content in the field of SCLC. The co-citation map of authors was analyzed by CiteSpace (**Figure 7B**). The authors with the top 10 centralities are shown in **Figure 7C**, suggesting that their works may serve as a bridge between various studies.

### Analysis of references

The top 10 SCLC-related publications with the most citations are shown in **Supplementary Table 1**. Local citation and global citation are often used to reflect the regional (In this data set) and global (In WoSCC) academic impact of the papers, respectively, and normalized citations processed by a special algorithm can effectively reduce the impact of publication time censoring bias associated with the measurement of citations (27). It turned out that Leora, Horn, 2018, *New Engl J Med* (3) ranked first with 1021 global citations, followed by George, Julie, 2015, *Nature* (28) with 919 times and Martin, Peifer, 2012, *Nat Genet* (26) with 827 times. Leora, Horn, 2018, *New Engl J Med* (3) (53.53 times), Carl M Gay, 2021, *Cancer Cell* (29) (33.86 times), and Luis Paz-Ares, 2019, *Lancet* (4) (32.55 times) were the top three articles in terms of normalized global citations. The citations of Carl M Gay, 2021, *Cancer Cell* (29) are at a disadvantage compared with other high cited literature published earlier due to the short publication period, which was eliminated with the usage of the normalized citations.

**FIGURE 8:**
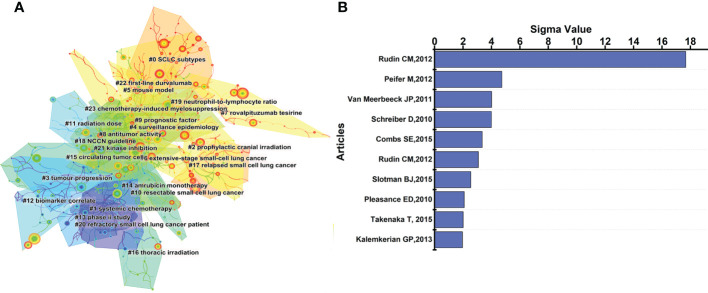
**(A)** Co-citation relationships between references. The figure shows 24 color blocks representing 24 clusters, each composed of articles on the same topic. **(B)** The top 10 articles of Sigma value in the reference co-citation map. Rudin CM, NAT GENET, 2012 has a much higher sigma value than other articles, proving that it is greatly innovative and valuable.

**FIGURE 9:**
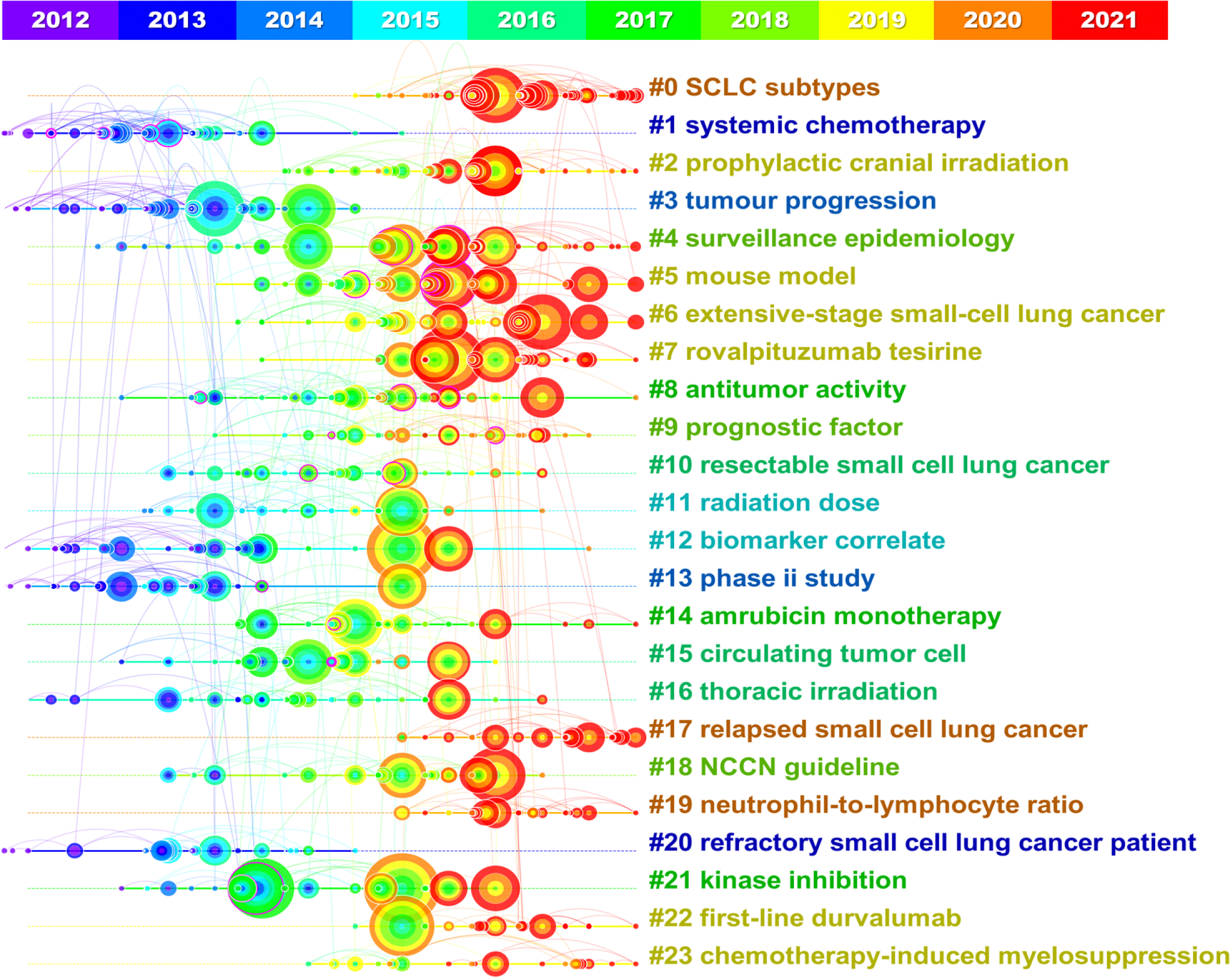
Timeline view of co-citation reference. The timeline from left to right represents the time evolution from 2012-2021. Nodes composed of different colors represent citations. The greater the node is, the larger the citations’ quantity is. The color of the node corresponds to the cited time for the time band. The timeline view visually shows the rise and fall of each area.

The reference co-citation relationship was visualized in a co-citation network (**Figure 8A**). The leading 10 articles with Sigma values in the co-citation graph are shown in **Figure 8B**, where the top 3 are: Rudin, Charles M., *Nat Genet*, 2012 (30) (Sigma=17.67), Martin, Peifer, *Nat Genet*, 2012 (26) (Sigma=4.73), Jan P van Meerbeeck, *Lancet*, 2011 (31) (Sigma=4.01). As illustrated in **Figure 8A**, all the references could be divided into 24 clusters. The timeline represents the time span from 2012 to 2021 from left to right, while the node represents the citations. The citation time can refer to the color band on the graph. These clusters may contain a major portion of the SCLC research over the past 10 years. Q value can evaluate the structure of cluster analysis, while S value can represent the homogeneity of each cluster. It is generally believed that Q > 0.3 mean the cluster structure is significant, while S > 0.5 are considered to be reasonable. In this study, Q value = 0.8501 and S value = 0.9489, indicating that the results of this study are convincing. **Figure 9** showed the development of the 24 clusters over 10 years, with the largest cluster #0 SCLC subtypes peaked in 2019 and has been continuously studied since then. However, the second largest cluster #1 systemic chemotherapy showed a decreasing trend year by year, indicating that the heat of this field gradually decreased. Based on the above results, Leora, Horn, 2018, *New Engl J Med* (4) and Rudin, Charles M., *Nat Genet*,2012 (30) can be defined as classic literature according to the number of citations, normalized citations and Sigma value.

### Analysis of keywords

**FIGURE 10:**
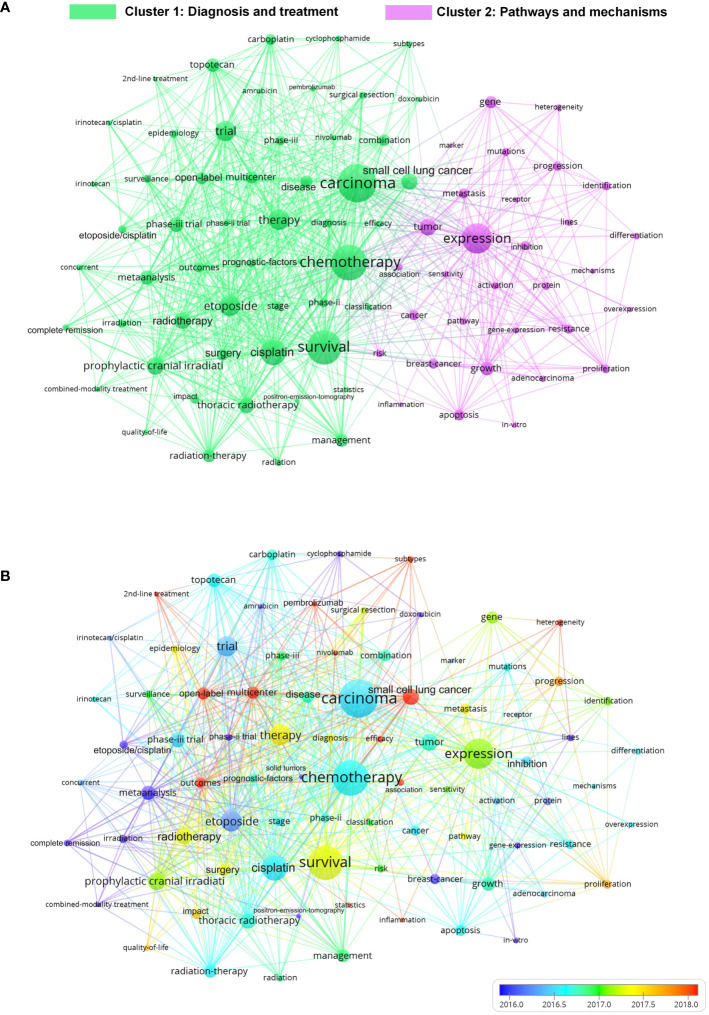
**(A)** Co-occurrence map of keywords on SCLC research generated by the VOS viewer. The keywords in the figure are divided into 2 distinct clusters: Cluster1: Diagnosis and treatment; Cluster 2: ;Pathways and mechanism. **(B)** Overlay map of co-occurrence keywords. The redder node in the figure represents the latest time of appearance. That is, the future hotspots we are looking for. .

There are 3,584 keywords selected by authors and 3,520 calculated keywords plus in SCLC articles in the past decade. Keywords plus refers to the high-frequency words extracted from abstracts, which was employed to eliminate the influence of the keywords’ limitations, greatly enhancing the possibility of screening research frontiers through bibliometric analysis. As displayed in **Figure 10A**, the results of co-occurrence analysis of keywords plus revealed that the key keywords with the most co-occurrence are chemotherapy, carcinoma, survival, therapy, expression, etc, which may be the cornerstone of the research on SCLC and the jumping-off point of many researchers. Besides, it is noteworthy to mention that the keywords in the co-occurrence graph can be divided into two clusters, namely Diagnosis and treatment was Cluster 1 and Pathways and mechanism was Cluster 2, which indicate that clinical and basic research are two major research directions of SCLC. The 20 keywords with the largest TLS in the co-occurrence map are shown in **Supplementary Table 2**. The overlay map of keyword co-occurrence analysis further revealed the timeliness of the keyword’s co-occurrence (**Figure 10B**), where the red nodes represent the latest average appearing year (AAY) which may be the future hotspot of SCLC research as shown in the color band in the lower-right corner of **Figure 10B**. “Subtypes” and “pembrolizumab” exhibit the latest AAY of 2020.12 and 2019.14 respectively in Cluster 1, while “heterogeneity”(AAY=2019.10) is the latest keyword in Cluster 2, which are all considered as potential frontiers of coming SCLC research.

## Discussion

In this era of the information explosion, it is challenging for researchers to keep track of the development of a field. As a scientific and creative tool, bibliometrics analysis enables researchers to overview the past, present, and even future of a field, which provides us with concise information about the development trend of research in a certain field in the past few decades and offers an important reference for researchers to guide future research (32). Previous bibliometric studies related to lung cancer focused on the application of cisplatin in SCLC (25), 100 articles with the highest citation in lung cancer (33), lung nodule study (34), and lobectomy versus segmentectomy (35), etc. In this study, bibliometric analysis and visualization of SCLC articles in the WoSCC database over the past 10 years were performed. SCLC is defined as a recalcitrant disease with rapid breakthroughs in the research progress, making it necessary to summarize the research history in this period by bibliometric methods. Besides, a 10-year bibliometric study interval is needed to ensure the timeliness of the study and the representativeness of summarizing the research status and trends. The atlas produced by the bibliometric method in the study is also called mapping knowledge domains (MKD) (36), allowing the visualization of relationship between targets through simple maps instead of numeric indicators. A query box (**Box 1**) was designed due to the fact that the errors in the input data of off-the-shelf tools such as CiteSpace and VOS viewer will lead to low precision, low fidelity, and even erroneous interpretation and conclusions (37).

### Trends of the SCLC research domain

The number of SCLC-related articles has risen steadily over the past decade, accompanied by substantial citations. As the number of published articles increases year by year, the average total citations per document decreases along. And the average total citation per year also shows a downward trend after a brief increase. A total of 2,312 articles with 44,812 citations and 400,090 references were extracted in this study. Moreover, 12,352 authors from 2,996 organizations published SCLC articles in 529 journals between 2012 and 2021.

Here is the trend analysis of the countries with the top three number of articles on SCLC, whose high involvement in SCLC research may be attributed to the high incidence of SCLC: (i). China: China holds the largest number of SCLC articles with the fastest growth rate of annual publications, indicating the increasing number of Chinese researchers on SCLC. However, the citation rate of most Chinese articles is not high, resulting in a low average number of citations per article. This phenomenon reminds Chinese researchers to concentrate on high-impact studies rather than pursuing the amount of papers. In this field, China cooperates closely with the United States, whereas there is less contact with countries such as Japan, Korea, and Europe, which are also authorities in the SCLC domain. The importance of collaboration is particularly noteworthy in the face of such data. (ii). The United States: The United States ranked second in the number of SCLC articles with far superior citations, illustrating the high impact of the United States researches in SCLC research. The high proportion of multiple-country publications suggested that researchers in the United States focus on communication and collaboration in scientific research. Notably, the number of SCLC publications of the United States had not increased significantly in recent years, for which the breadth of research needs to be expanded. (iii). Japan: Japan ranks third in the world both in terms of the number of publications and total citations in the field of SCLC with few multiple-country publications, which suggested the lack joint research of Japan with other countries. The relatively slow growth rate of SCLC papers in Japan and even a decrease in some years, indicates that SCLC research in Japan is on a downward trajectory over the past decade and more worthwhile studies are needed to be conducted.

As for organizations, the University of Texas MD Anderson Cancer Center, Memorial Sloan Kettering Cancer Center, Shandong University, and Chinese Academy of Medical Sciences are the most active organizations in the SCLC field, which are mainly distributed in North America, Europe, and Asia, with the cooperation between research organizations still being regional according to the co-authorship map (**Figure 4A**). Regarding journals, *Lung Cancer* and *the Journal of Thoracic Oncology* made significant contributions to SCLC research, as many outstanding articles have been published by the two journals, some journals excluded by SCI also rank well by the bibliometric analysis, which reminds researchers to keep their eyes polished when choosing a journal to publish their findings. For authors, the results of this study point out that the researches of several authors including Rudin, Charles M., Sage, Julien, and Leora, Horn in SCLC field deserve to be investigated. An author’s research achievements can be comprehensively evaluated by the number of papers, H-index, and citations, which is one of the advantages of bibliometric analysis. Only 27 articles in this study were written by a single author, indicating that the majority of academic research was still carried out by cohorts rather than individuals.

### Current and future hotspots of the SCLC research domain

The latest of the top 10 articles cited was published in 2019, while the earliest was published in 2012, where Leora, Horn, 2018, *New Engl J Med* (3) and Rudin, Charles M., Nat Genet, 2012 (30) were defined as classic literature by the bibliometric method, indicated their high research value. The status of immunotherapy in SCLC was established by Leora, Horn, 2018, New Engl J Med (3) and ushered in the era of immunotherapy. Besides, comprehensive genomic analysis was performed in Rudin, Charles M., Nat Genet, 2012 (30), and it was concluded that SOX2 was a frequently amplified gene in SCLC, which laid a foundation for the precision therapy of SCLC. With normalized citations, Carl M Gay, 2021, *Cancer Cell* (29) can also be considered as high-cited articles which may receive higher citations in the future. Among the 10 highly cited articles listed in **Supplementary Table 1**, 5 manuscripts focused on immunotherapy, 3 papers centered on genomics, and 2 articles concentrated on circulating tumor cells, where clinical trials related to immunotherapy have attracted considerable attention in particular. As shown in **Figure 9**, 0# SCLC subtypes, #5 mouse model, #6 extensive-stage small-cell lung cancer, and #17 relapsed small cell lung cancer were still highly cited in 2021, which might be the representatives of the hot spots in current research. The 4 latest AAY keywords extracted in **Figure 10B** are “pembrolizumab,” “nivolumab,” “heterogeneity” and “subtypes”, which can be considered as the direction of future SCLC research. Based on the above results, future studies on SCLC will focus on (i). Heterogeneity & Subtypes (ii). Immunotherapy:

(i). Heterogeneity & Subtypes: SCLC has always been regarded as a homogeneous disease and was treated indiscriminately as a whole (38), yet the recognition of SCLC has deepened with the advancement of detection technology and the development of bioinformatics (28, 39, 40). Recent studies have shown that SCLC is markedly heterogeneous at the molecular level. Inactivation of tumor suppressor genes like TP53 and RB1 are common in almost all cases of SCLC (28). Researchers have also changed their minds about SCLC subtypes from the traditional “classic” and “variant” types to “NE” and “non-NE” phenotypes, which were then shifted to subtypes defined by major transcription regulatory factors. Rudin et al. divided SCLC into SCLC-A, N, P, and Y subtypes according to the differences in the expression of transcription factors including ASCL1, NeuroD1, POU2F3, and YAP1 in 2019 (41). Afterward, Rudin et al. found a new subgroup of high-expression PLCG2 distinct from the above-mentioned type 4, which exhibited stem cell-like characteristics and promoted metastasis, in relation to profibrotic and immunosuppressive mononuclear/macrophage enrichment in tumors (42). However, subsequent studies identified that YAP1 is probably not the key transcription factor that precisely explains the SCLC subtype, and further discovered and defined SCLC-inflamed or SCLC-I subtype rich in immune and inflammatory phenotypes, where SCLC-I subtype in the latest SCLC subtype was inflammatory and sensitive to immunotherapy, while other subtypes were not (29). This research result points to a new exploration direction for prospective precision therapy, which is to select appropriate treatment methods for different subtypes (43). The investigation of molecular types and characteristics of different subtypes of SCLC will facilitate the comprehension of the biological characteristics of varied subtypes of SCLC and determine the differences, commonalities, and mutual transformation ability between distinctive subtypes (44). Finally, the key factors affecting the occurrence, development, and metastasis of different subtypes of SCLC will be found, which will assist physicians in the diagnosis and further optimize treatment options for SCLC patients in clinical applications, thereby prolonging the survival time and improving the life quality of patients suffering from SCLC (45).

(ii). Immunotherapy: In recent years, platinum-based chemotherapy has been the standard treatment for SCLC in limited and extensive stages (46, 47). However, SCLC patients are susceptible to chemotherapy resistance, recurrence, and metastasis (48), which leaves patients with an average survival time around 8 months to 13 months and a five-year survival rate less than 6%-7% (49). The emergence of immune checkpoint inhibitors (ICIs), has enabled significant achievements in various fields of cancer (50), whose applications in SCLC have also been explored over the past years. IMPOWER-133 study (3) confirmed that the addition of atezolizumab to standard chemotherapy can extend the overall survival (OS) of ES-SCLC patients by 2 months and reduce the risk of death by 30%, which also became the first phase III clinical trial in more than 30 years with significant OS improvement achieved in the first-line treatment of ES-SCLC and defined new criteria for first-line treatment of ES-SCLC. In the CASPIAN study (4), durvalumab combined with chemotherapy attained an OS of 13.0 months, which was also approved as the indication of ES-SCLC first-line treatment. Based on the results of the KEYNOTE-028 (51) and KEYNOTE-158 (51) studies, pembrolizumab can benefit some SCLC patients. Moreover, the CTLA-4 inhibitor Ipilimumab was the first SCLC immune checkpoint inhibitor to be evaluated prospectively (52). The results of CheckMate-032 demonstrated the efficacy and tolerability of Nivolumab in combination with ipilimumab for three-line therapy of recurrent SCLC (53). In addition, as *in vivo* delivery of ICIs remains a challenge, the combination of ICIs with drug-loaded nanoparticles has become a recent research focus, with photodynamic and thermal nanoparticle-enhanced therapies receiving the most attention (54). In conclusion, for the first time in decades, significant advances have been made in the SCLC field, with immune checkpoint inhibitors extending the survival of patients with ES-SCLC (5, 6, 55). However, the intended population that can benefit from immunotherapy remains restricted, and the most appropriate time to implement immunotherapy during the process of treatment is yet unclear, which needs to be confirmed by further clinical trials (2, 56).

## Limitations

A few SCLC articles were not covered due to the strict search formula designed to eliminate the influence of other types of lung cancer articles on the results. 2. Owing to the wide range of SCLC research, there may be an issue of overgeneralization 3. All data generated by the software are subjected to errors, and some results need to be identified by the experience of the researcher. 4. In this study, only one database was used, and multiple databases would be required for verification and supplementation in the future.

## Advantages

The accuracy was improved as both researchers rigorously tested multiple research formulas before finally selecting the optimal one that covered the most publications with all of them being SCLC-related articles. 2. The time frame for this study was the decade since SCLC was defined as recalcitrant cancer, during which a research boom occurred compared to the dormancy in the past decades, causing the necessity to carry out bibliometric analysis to summarize and analyze the foundation and frontiers of SCLC research so as to provide a guidance and reference for future investigations.

### Conclusions

At present, this is the first study to analyze the research trends of SCLC (2012–2021) by bibliometric methods. This study can provide a research basis for relevant researchers, fill the research gap, identify research hot spots and provide the readers with a more comprehensive understanding of SCLC. The research on SCLC has shown an upward trend in both the number of articles and citations in this decade, with China, the United States, and Japan making significant contributions. Nevertheless, *via* a multi-level analysis, it can be seen that cooperation still needs to be strengthened, especially among international organizations. The University of Texas MD Anderson Cancer Center, United States was the most prolific organization. The most productive and major source was *Lung Cancer* and *the Journal of Thoracic Oncology.* The author Rudin, Charles M. from the Memorial Sloan Kettering Cancer Center has the maximum number of citations and articles in SCLC domain. According to citation analysis and sigma value detection, the class literature was Leora, Horn, 2018, *New Engl J Med* (3) titled “First-line atezolizumab plus chemotherapy in extensive-stage small-cell lung cancer” in “*the New England Journal of Medicine*” and Rudin, Charles M., *Nat Genet*, 2012 (30) titled “Comprehensive genomic analysis identifies SOX2 as a frequently amplified gene in small-cell lung cancer” in “*Nature Genetics*”. The analysis of references and keywords revealed that the heat of chemotherapy gradually decreased, while the research related to immunotherapy is gaining popularity. Besides, researchers had also carried out many explorations on the heterogeneity of SCLC through genomics analysis. Therefore, this study predicts that “heterogeneity & subtypes” and “immunotherapy” may become the hot spots for future research, and the precision therapy of different subtypes of SCLC, the application of ICIs in SCLC treatment, as well as the progress of clinical trials will be the spotlight. Undoubtedly, the SCLC domain will attract ever-increasing numbers of researchers, and more valuable meaningful research will be further performed for the benefit of patients.

## Data availability statement

Publicly available datasets were analyzed in this study. This data can be found here: https://wcs.webofknowledge.com.

## Author contributions

KW and HZ: conceived the study and organized the retracted data. XLi and YD: made charts. JL and XLiu: wrote the manuscript. SS, ZW and DS: revised and reviewed the manuscript. All authors contributed to the article and approved the submitted version

## Funding

This study was supported by the Natural Funding Project of Tianjin Science and Technology Bureau (No. 20JCYBJC01350), Tianjin Health Science and Technology Project Key Projects (ZD20023) and Tianjin Key Medical Discipline (Specialty) Construction Project (TJYXZDXK-018A).

## Acknowledgment

The authors thank Yaping Zhang for help in language polishing.

## Conflict of interest

The authors declare that the research was conducted in the absence of any commercial or financial relationships that could be construed as a potential conflict of interest.

## Publisher’s note

All claims expressed in this article are solely those of the authors and do not necessarily represent those of their affiliated organizations, or those of the publisher, the editors and the reviewers. Any product that may be evaluated in this article, or claim that may be made by its manufacturer, is not guaranteed or endorsed by the publisher.
