# Supplementary material for: Bibliometric analysis of global research trends on small-cell lung cancer (2012–2021)
Source: Front Oncol. 2022 Oct 6;12:955259. doi: 10.3389/fonc.2022.955259 (PMC9583519; doi:10.3389/fonc.2022.955259)
Supplement: Supplementary file 1 [file DataSheet_1.docx]

**SUPPLEMENTARY TABLE 1 |** The top 10 SCLC-related publications with the most citations.

| **Rank** | **Articles** | **Year** | **Journal** | **First Author** | **GC** | **NGC** | **LC** | **NLC** | **IF 2020** |
| --- | --- | --- | --- | --- | --- | --- | --- | --- | --- |
| 1 | First-line atezolizumab plus chemotherapy in extensive-stage small-cell lung cancer | 2018 | NEW ENGL J MED | Leora, Horn | 1021 | 53.53 | 273 | 63.98 | 91.245 |
| 2 | Comprehensive genomic profiles of small cell lung cancer | 2015 | NATURE | George, Julie | 919 | 29.98 | 242 | 32.77 | 49.962 |
| 3 | Integrative genome analyses identify key somatic driver mutations of small-cell lung cancer | 2012 | NATURE GENETICS | Martin, Peifer | 827 | 20.38 | 197 | 22.35 | 38.33 |
| 4 | Nivolumab alone and nivolumab plus ipilimumab in recurrent small-cell lung cancer (CheckMate 032): a multicenter, open-label, phase 1/2 trial | 2016 | LANCET ONCOLOGY | Scott J Antonia | 740 | 28.92 | 132 | 26.14 | 41.316 |
| 5 | Comprehensive genomic analysis identifies SOX2 as a frequently amplified gene in small-cell lung cancer | 2012 | NATURE GENETICS | Charles M Rudin | 638 | 15.73 | 155 | 17.59 | 38.33 |
| 6 | Clinical significance and molecular characteristics of circulating tumor cells and circulating tumor microemboli in patients with small-cell lung cancer | 2012 | JOURNAL OF CLINICAL ONCOLOGY | Jian-Mei Hou | 551 | 13.58 | 52 | 5.90 | 44.544 |
| 7 | Durvalumab plus platinum–etoposide versus platinum–etoposide in first-line treatment of extensive-stage small-cell lung cancer (CASPIAN): a randomised, controlled, open-label, phase 3 trial | 2019 | LANCET | Luis Paz-Ares | 448 | 32.55 | 147 | 47.22 | 79.321 |
| 8 | Tumorigenicity and genetic profiling of circulating tumor cells in small-cell lung cancer | 2014 | NATURE MEDICINE | Cassandra L Hodgkinson | 446 | 15.12 | 46 | 7.78 | 53.44 |
| 9 | Tumor mutational burden and efficacy of nivolumab monotherapy and in combination with ipilimumab in small-cell lung cancer | 2018 | CANCER CELL | Matthew D. Hellmann | 405 | 21.23 | 45 | 10.55 | 31.743 |
| 10 | Ipilimumab in combination with paclitaxel and carboplatin as first-line therapy in extensive-disease-small-cell lung cancer: results from a randomized, double-blind, multicenter phase 2 trial† | 2013 | ANNALS OF ONCOLOGY | M. Reck | 401 | 13.58 | 30 | 4.52 | 32.976 |

GC: Global Citations, NGC: Normalized Global Citations, LC: Local Citations, NLC: Normalized Local Citations

**SUPPLEMENTARY TABLE 2 |** The top 20 keywords with the highest TLS.

| **Rank** | **Keyword** | **Occurrences** | **TLS** | **Rank** | **Keyword** | **Occurrences** | **TLS** |
| --- | --- | --- | --- | --- | --- | --- | --- |
| 1 | chemotherapy | 400 | 1628 | 11 | meta-analysis | 117 | 599 |
| 2 | carcinoma | 439 | 1509 | 12 | radiotherapy | 138 | 539 |
| 3 | survival | 372 | 1359 | 13 | phase-iii trial | 123 | 505 |
| 4 | cisplatin | 259 | 1221 | 14 | radiation-therapy | 106 | 491 |
| 5 | etoposide | 201 | 1025 | 15 | small cell lung cancer | 142 | 456 |
| 6 | expression | 316 | 857 | 16 | topotecan | 115 | 434 |
| 7 | trial | 187 | 767 | 17 | multicenter | 100 | 432 |
| 8 | prophylactic cranial irradiation | 156 | 699 | 18 | surgery | 93 | 411 |
| 9 | thoracic radiotherapy | 133 | 691 | 19 | tumor | 149 | 406 |
| 10 | therapy | 193 | 681 | 20 | combination | 92 | 399 |

**
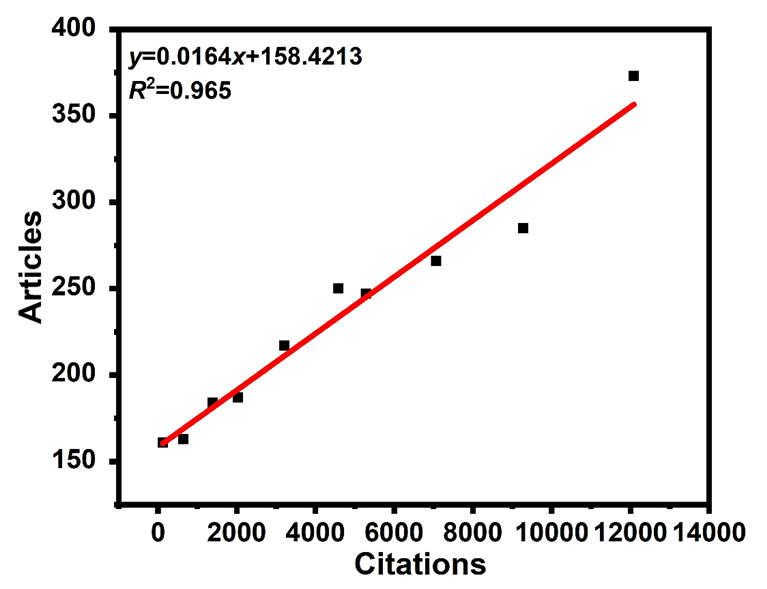
**

**SUPPLEMENTARY FIGURE 1 |** A linear regression about the annual growth of article and citation number.

**
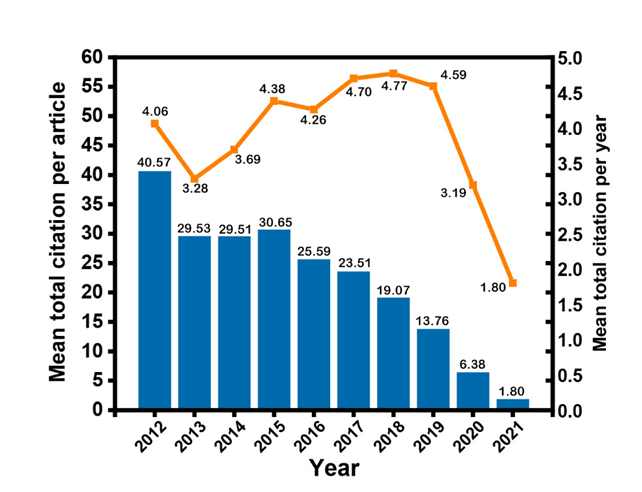
**

**SUPPLEMENTARY FIGURE 2 |** Distribution of average total citations of documents yearly.
